# Exploring the roles of fecundity-related long non-coding RNAs and mRNAs in the adrenal glands of small-tailed Han Sheep

**DOI:** 10.1186/s12863-020-00850-6

**Published:** 2020-04-06

**Authors:** Qing Xia, Qiuling Li, Shangquan Gan, Xiaofei Guo, Xiaosheng Zhang, Jinlong Zhang, Mingxing Chu

**Affiliations:** 1 Key Laboratory of Animal Genetics and Breeding and Reproduction of the Ministry of Agriculture and Rural Affairs, Institute of Animal Science, Chinese Academy of Agricultural Sciences, Beijing, 100193 P. R. China; 2 College of Life Sciences, Langfang Normal University, Langfang, 065000 P.R. China; 3 State Key Laboratory of Sheep Genetic Improvement and Healthy Production, Xinjiang Academy of Agricultural and Reclamation Sciences, Shihezi, 832000 P. R. China; 4 Tianjin Institute of Animal Sciences, Tianjin, 300381 P. R. China

**Keywords:** LncRNAs, Follicle phase, Luteal phase, Small-tailed Han sheep, Adrenal

## Abstract

**Background:**

Long non-coding RNAs (lncRNAs) can play important roles in uterine and ovarian functions. However, little researches have been done on the role of lncRNAs in the adrenal gland of sheep. Herein, RNA sequencing was used to compare and analyze gene expressions in adrenal tissues between follicular phases and luteal phases in *FecB*^*BB*^ (MM) and *FecB*^*++*^ (WW) sheep, respectively, and differentially expressed lncRNAs and genes associated with reproduction were identified.

**Results:**

In MM sheep, 38 lncRNAs and 545 mRNAs were differentially expressed in the adrenal gland between the luteal and follicular phases; In WW sheep, 513 differentially expressed lncRNAs and 2481 mRNAs were identified. Gene Ontology and Kyoto Encyclopedia of Genes and Genomes enrichment analyses indicated that differentially expressed lncRNAs and their target genes are mainly involved in the circadian rhythm, the mitogen activated protein kinase, thyroid, ovarian steroidogenesis and transforming growth factor beta signaling pathways. Differentially expressed lncRNAs can regulate reproduction by modulating genes involved in these signaling pathways and biological processes. Specifically, *XLOC_254761*, *XLOC_357966*, *105,614,839* and *XLOC_212877* targeting *CREB1*, *PER3*, *SMAD1* and *TGFBR2*, respectively, appear to play key regulatory roles.

**Conclusion:**

These results broaden our understanding of lncRNAs in adrenal gland of sheep and provide new insights into the molecular mechanisms underlying sheep reproduction.

## Background

Small-tailed Han sheep are famous for their high fertility, precocious puberty, good fur quality and tall body shape in China [1]. The behavior of estrus and mate in Small-tailed Han sheep appear year-round. The lambing rate of primiparous ewes is about 200%, and in produced ewes is higher than 250% [1]. *Fec*^*BB*^ mutations have huge economic benefits in production, which can significantly increase the number of ovulation and lambs in sheep. According to the previous research report of this team, all three genotypes of *FecB* (*FecB*^*BB*^, *FecB*^*B+*^ and *FecB*^*++*^) are distributed in Small-tailed Han sheep, and there is a significant correlation between the three genotypes of *FecB* and the lamb size of ewes [2]. Therefore, Small-tailed Han sheep can be used as an ideal animal model to study the molecular mechanism of *FecB* gene regulation of reproductive traits.

In recent years, there have arisen many methods for identifying differentially expressed candidate long noncoding RNAs (lncRNAs) and genes using transcriptome sequencing. lncRNAs are RNAs of > 200 nucleotides in length. Studies have shown that lncRNAs play important roles in many life activities, such as dose-compensation effects, epigenetic regulation, cell cycle regulation, cell differentiation and regulation, and have become a hot research topic in genetics [3–6]. For example, Yang et al. (2018) identified differentially expressed lncRNAs and mRNAs in the testes of prepubertal and mature rams that were enriched in spermatogenetic and male gonadal developmental signaling pathways [5]. Zheng (2019) analyzed the pituitaries of Hu sheep with high and low fertility and found 57 differentially expressed lncRNAs and 298 differentially expressed mRNAs [6]. Miao et al. (2016, 2017) analyzed the ovaries of Small-tailed Han and Dorset sheep strains and found that differentially expressed lncRNAs were significantly enriched in the oxytocin signaling pathway. Methylation of lncRNAs might contribute to improving the reproduction of Small-tailed Han sheep [3, 4]. Feng (2018) identified 76 differentially expressed mRNAs and 5 differentially expressed lncRNAs by analyzing the ovaries of Hu sheep with high and low reproduction rates [7]. These studies showed that lncRNAs in the pituitary and ovaries of sheep have regulatory functions in reproduction. It is known that the sheep adrenal gland also has an impact on reproduction [8–12], but studies on the functions of lncRNAs in this organ are limited.

In this study, the differentially expressed genes in the adrenal gland between Small-tailed Han sheep with *FecB*^*BB*^ (MM) and Small-tailed Han sheep with *FecB*^*++*^ (WW) (hitherto simply MM and WW sheep) were analyzed using RNA sequencing (RNA-Seq). The molecular mechanisms of differentially expressed lncRNAs and genes in the adrenal gland related to reproduction were subjected to a preliminary exploration. Our results provide an effective theoretical basis for studying the molecular mechanisms by which lncRNAs regulate sheep reproduction.

## Results

### Transcript assembly and quality control

The RNA-seq data of 12 samples were subjected to quality control, and the results are shown in Table 1. The clean reads for each sample ranged from 80 to 100 million and the Q30 values ranged from 92.50 to 95.30%. About 89.47–91.63% of the clean reads were mapped to the sheep reference genome, and about 80% were mapped uniquely.

**Table 1:** Summary of quality data after quality control

Sample	Raw read s	Clean read s	Clean reads rate(%)	Q30(%)	Total mapped rea ds	Uniquely mapped rea ds
MF1	121,899,310	118,357,160	97.09	95.30	90.83%	82.16%
MF2	100,514,168	97,878,852	97.38	94.12	91.63%	83.62%
MF3	93,565,822	91,784,982	98.10	94.30	90.28%	84.74%
MF1	89,881,752	86,058,280	95.75	92.68	89.61%	85.02%
MF2	91,763,718	88,508,870	96.45	92.79	90.52%	82.55%
MF3	88,087,492	84,832,940	96.31	92.77	89.98%	82.94%
MF1	109,076,604	105,978,806	97.16	93.94	89.76%	81.41%
MF2	106,524,486	101,591,104	95.37	93.01	89.47%	80.75%
MF3	89,866,984	86,416,914	96.16	92.50	89.87%	81.97%
MF1	87,611,256	85,783,998	97.91	92.96	91.46%	81.32%
MF2	102,807,796	99,286,834	96.58	93.28	89.95%	80.54%
MF3	102,733,338	99,271,932	96.63	92.65	90.76%	82.68%

Note: MF and ML represent follicular phase and luteal phase in MM sheep, respectively; WF and WL represent follicular phase and luteal phase in WW sheep, respectively

### LncRNA identification and characterization

**Fig. 1:**
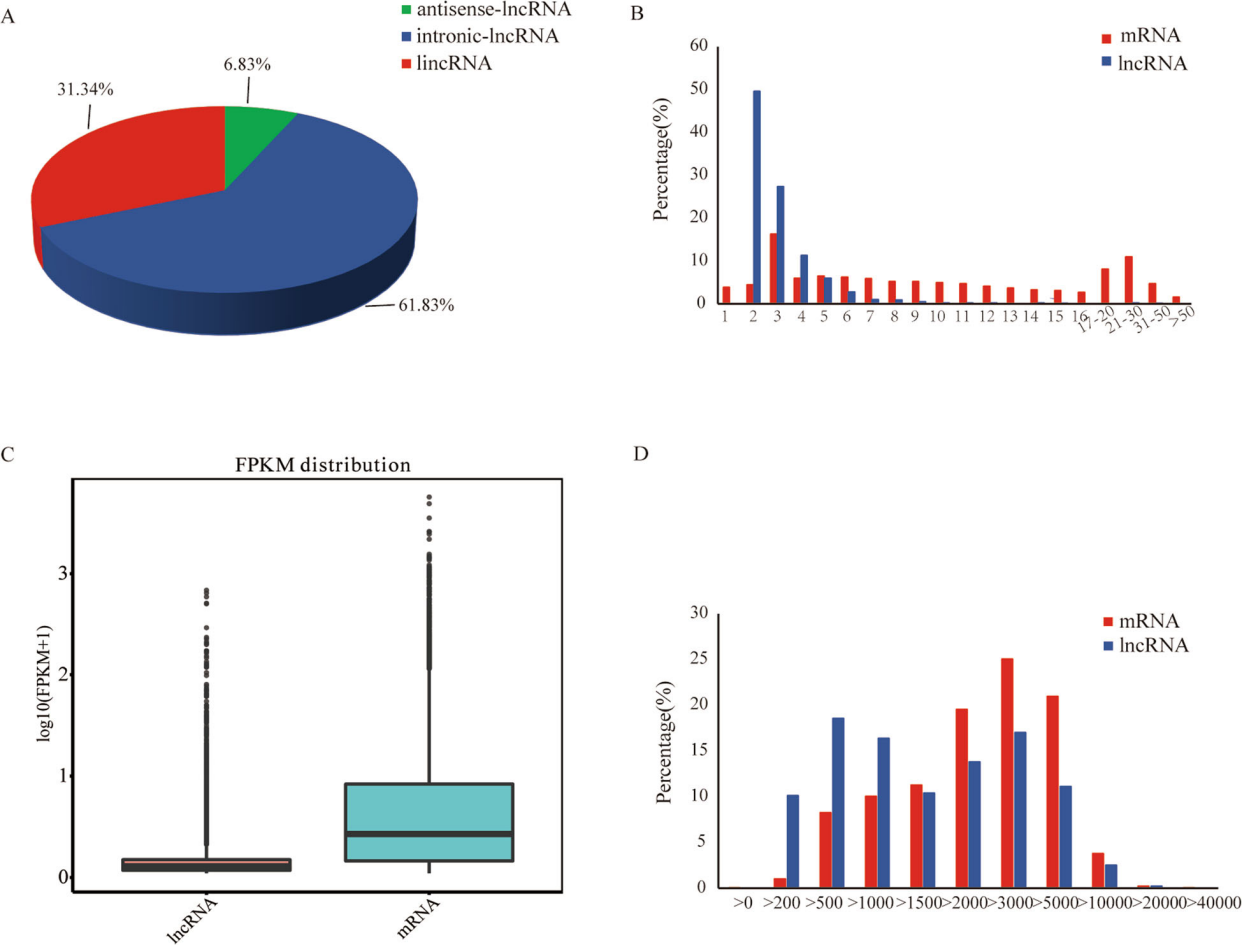
LncRNA characterization and gene expression. **a** Summary of lncRNAs types. **b** The exon number distributions of lncRNAs and mRNAs. **c** The expression levels of lncRNAs and mRNAs. **d** The length distribution of lncRNAs and mRNAs

A total of 17,201 candidate lncRNAs was identified, including 1174 anti-sense lncRNAs, 10,636 intronic lncRNAs and 5391 large intergenic (linc) RNAs (Fig. 1a). As shown in Fig. 1b, most lncRNAs had two exons: significantly fewer than the exons of mRNAs. The expression levels of mRNAs and lncRNAs were further analyzed according to FPKM values, and the boxplot (Fig. 1c) shows that the expression levels of mRNAs in the adrenal tissues were higher than lncRNAs. The distributions of lncRNAs and mRNAs lengths were consistent (Fig. 1d).

### Expression levels of genes and differentially expressed analysis

The Fig. 1c box plot shows that lncRNAs transcript expression levels were all lower than those of mRNAs in adrenal of both MM and WW Small-tailed Han sheep. Based on *P* values < 0.05, differentially expressed gene analysis of adrenal tissues showed that there were 15 lncRNAs upregulated and 23 downregulated in MM sheep; 279 mRNAs were upregulated and 266 downregulated in MM sheep (Supplemental Table S1). In WW sheep, 354 lncRNAs were upregulated and 159 downregulated; 1334 mRNAs were upregulated and 1147 downregulated (Supplemental Table S1 & S2).

### GO annotation and KEGG enrichment analysis of differentially expressed mRNAs

**Fig. 2:**
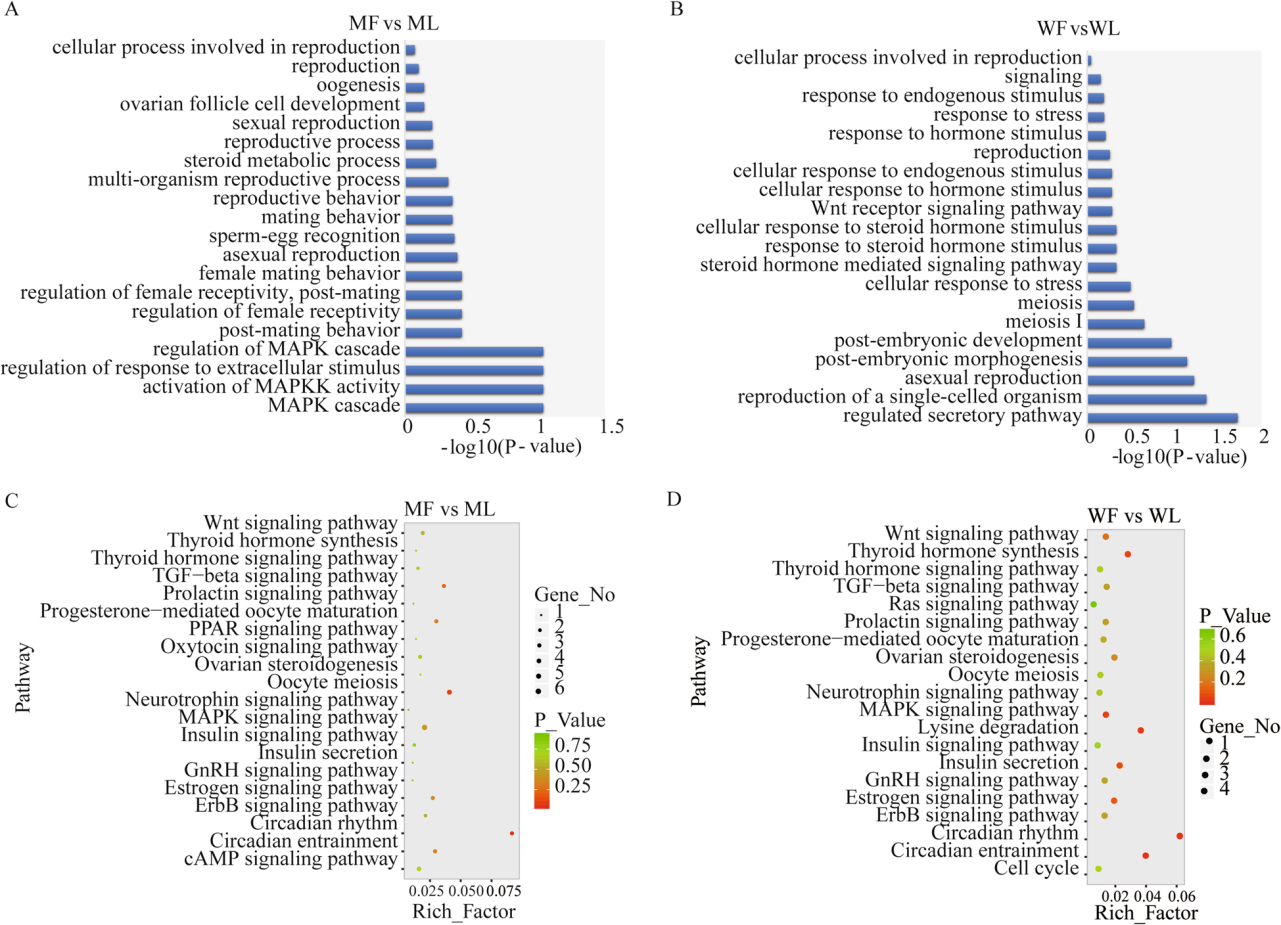
Go annotation and KEGG pathway enrichment analysis of differentially expressed mRNAs. **a** The differentially expressed mRNAs according to GO enrichment analysis in MM sheep. **b**The differentially expressed mRNAs according to GO enrichment analysis in WW sheep. **c** KEGG enrichment pathways for differentially expressed mRNAs are presented for the MM sheep. **d** KEGG enrichment pathways for differentially expressed mRNAs are presented for the WW sheep. Suffixes _F and _L refer to the follicular phase and luteal phase, respectively. Note: Rich_factor is defined as amount of differentially expressed genes enriched in the pathway/amount of all genes in background gene set

The GO enrichment analysis of all differentially expressed mRNAs showed that they were categorized into BP, CC, and MF. The differentially expressed mRNAs in MM and WW sheep associated with reproduction were mainly annotated in biological processes, and these were involved in reproduction processes, mating behavior, ovarian follicle cell development, the mitogen-activated protein kinase (*MAPK*) cascade and steroid metabolic processes in MM sheep (Fig. 2a, Supplemental Table S3). In WW sheep, these were involved in the *Wnt* receptor signaling pathway, meiosis, post-embryonic development, asexual reproduction and responses to steroid hormone stimuli (Fig. 2b, Supplemental Table S4).

The KEGG enrichment analysis showed that the majority of differentially expressed mRNAs were enriched in the same pathways in both the MM and WW sheep. The differentially expressed mRNAs were enriched in progesterone-mediated oocyte maturation, the *MAPK* signaling pathway, circadian rhythm, oocyte meiosis and insulin signaling pathway (Fig. 2c and d, Supplemental Table S5).

### GO annotation and KEGG enrichment analysis of target genes of lncRNAs

**Fig. 3:**
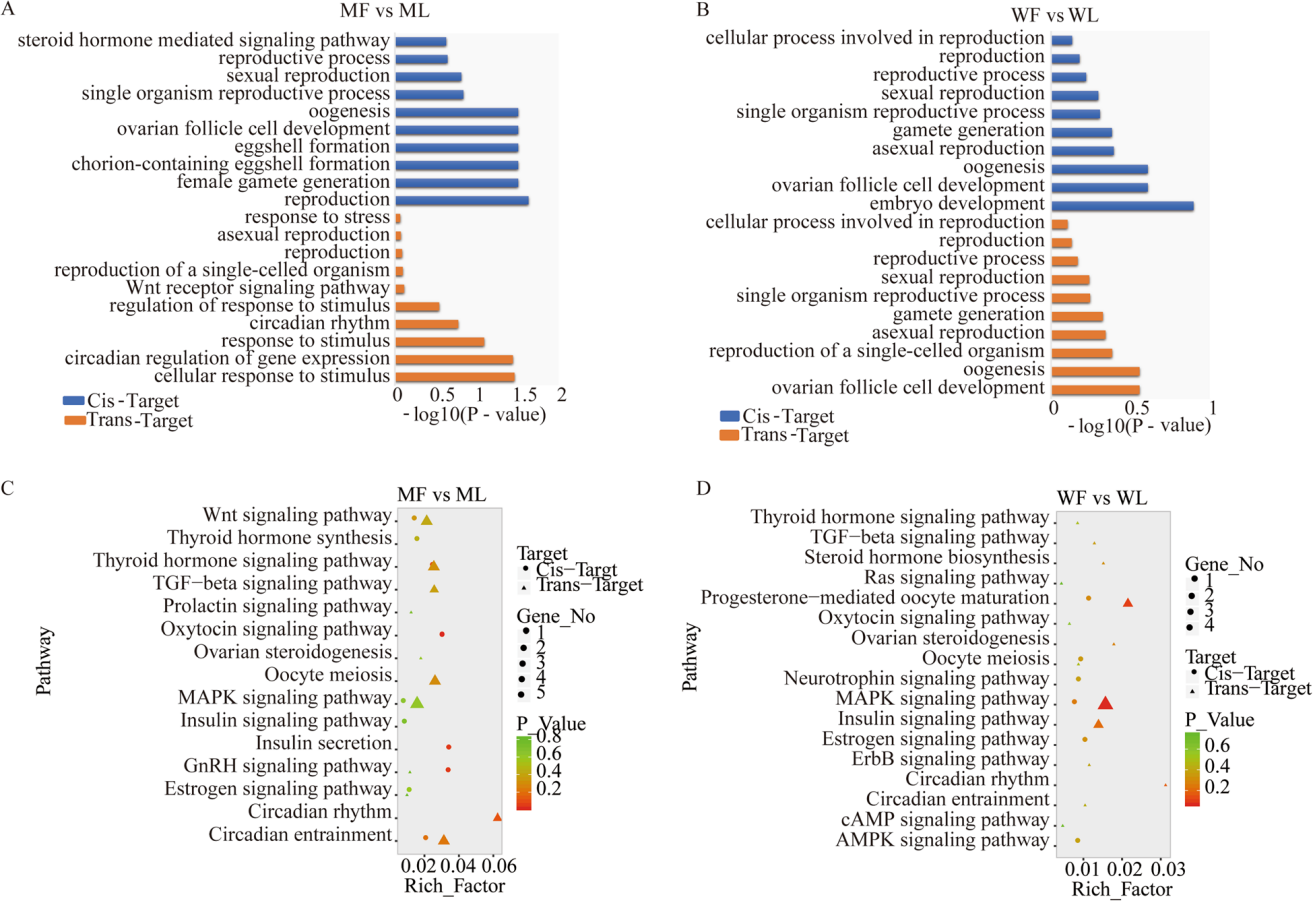
GO annotation and KEGG pathway analysis of differentially expressed lncRNA targets. **a**The differentially expressed lncRNA target gene GO enrichment analysis for MM sheep. **b**The differentially expressed lncRNA target gene GO enrichment analysis for WW sheep. **c** KEGG enrichment pathways for differentially expressed lncRNA targets are presented for MM sheep. **d** KEGG enrichment pathways for differentially expressed lncRNA targets are presented for WW sheep. Suffixes _F and _L refer to the follicular phase and luteal phase, respectively. Note: Rich_factor is defined as amount of differentially expressed genes enriched in the pathway/amount of all genes in background gene set

LncRNAs target genes were analyzed according to GO annotation and KEGG enrichment. As shown by the GO annotation results, lncRNA target genes associated with reproduction were also mainly annotated in biological processes in the MM and WW sheep. As shown in Fig. 3, both cis-target genes and trans-target genes associated with reproduction were enriched in biological processes in the MM type and WW sheep. In the MM sheep, cis-targets were mainly enriched for female gamete generation, reproduction and oogenesis, and trans-targets were enriched for the *Wnt* receptor signaling pathway, reproduction and circadian rhythm (Fig. 3a). Cis-targets and trans-targets were all mainly enriched for reproduction, gamete generation and ovarian follicle cell development (Fig. 3b).

The KEGG enrichment analysis associated with reproduction showed that the majority of cis-target genes and trans-target genes were enriched in the same pathways in MM and WW sheep (Fig. 3). In MM sheep, the lncRNA targets were assigned to 20 reproduction pathways, such as *Wnt* signaling pathway, transforming growth factor beta (*TGFβ*) signaling pathway, ovarian steroidogenesis, *MAPK* signaling pathway, circadian rhythm and other (Fig. 3c). The lncRNA targets were enriched in progesterone-mediated oocyte maturation, *MAPK* signaling pathway, circadian rhythm, oocyte meiosis and insulin signaling pathway (Fig. 3d; Supplementary Table S6).

### LncRNAs and mRNAs co-expression network analysis

**Fig. 4:**
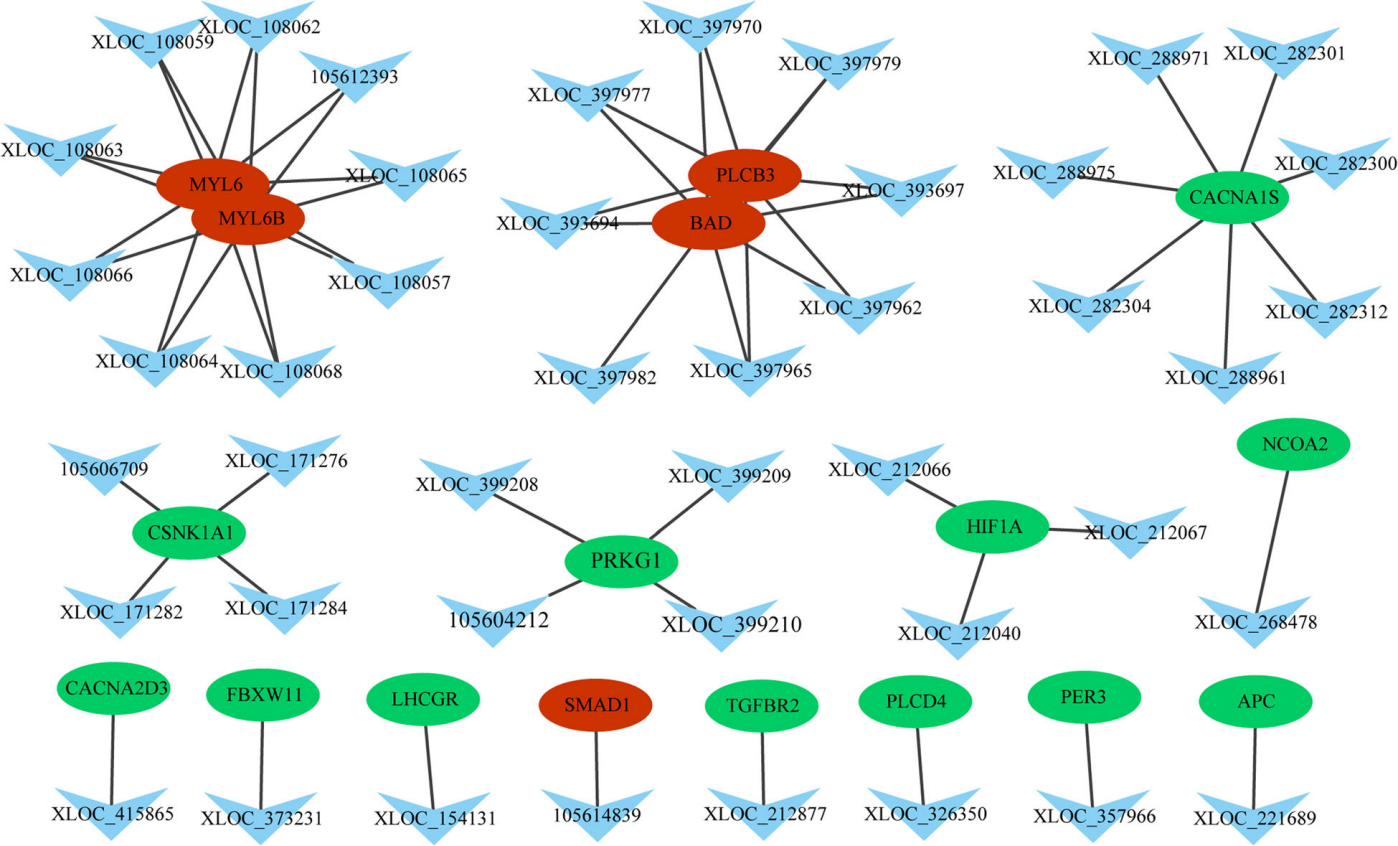
Network between differentially expressed lncRNAs and lncRNA targets in the adrenal glands of MM sheep. Note:The node of each triangle represents lncRNAs, and the circular nodes represent lncRNA target genes. Red nodes (ovals) represent upregulated genes and green nodes represent downregulated genes

**Fig. 5:**
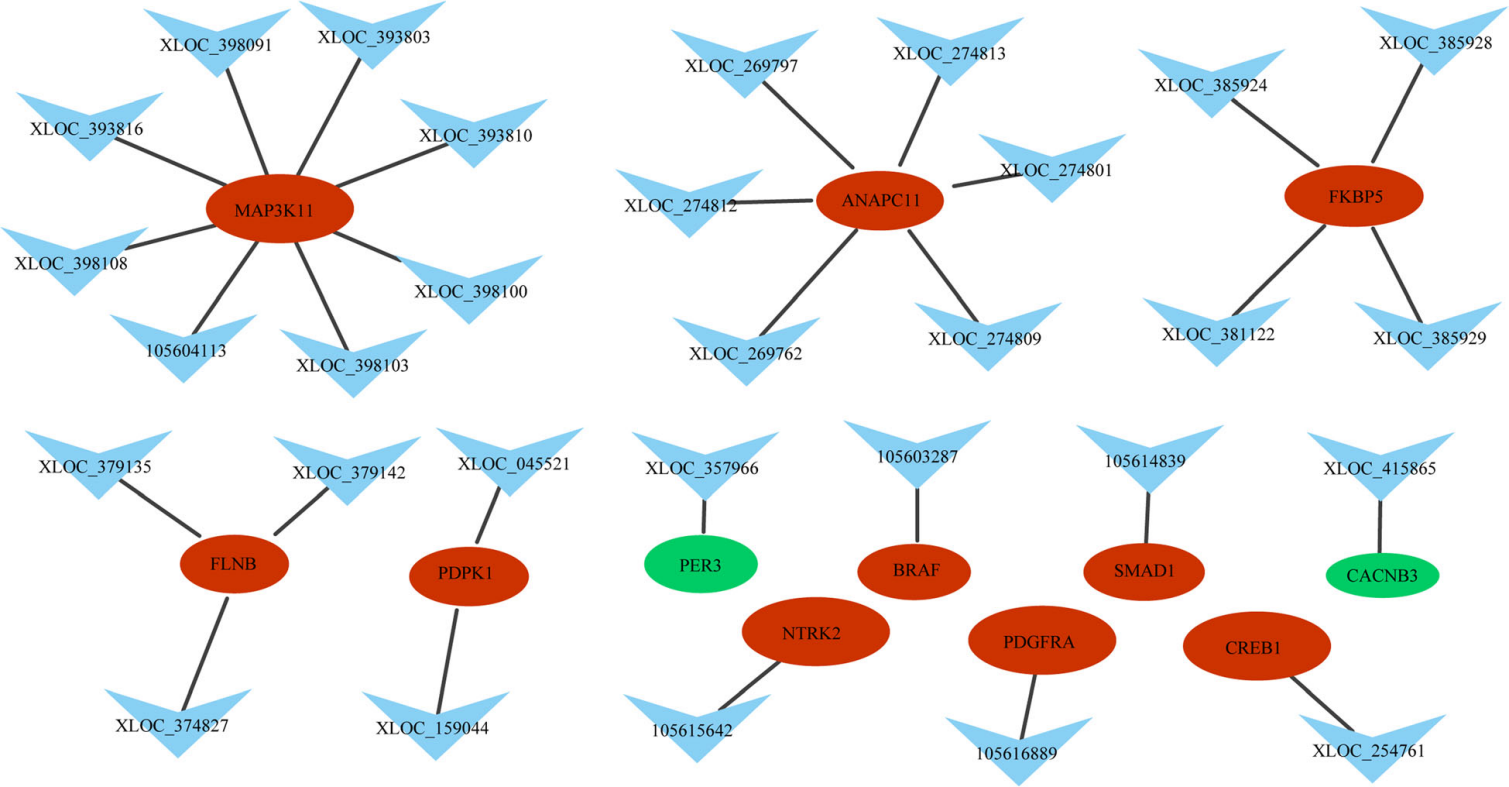
Network between differentially expressed lncRNAs and lncRNA targets in the adrenal glands of WW sheep. Note: the nodes of the triangles represent lncRNAs, and the circular nodes represent lncRNA target genes. Red ovals represent nodes for upregulated genes, green ovals represent nodes for downregulated genes

In the MM sheep, a lncRNA/mRNA co-expression network was constructed using 46 differentially expressed lncRNAs and 17 target genes involved in reproductive-related pathways. As shown in Fig. 4, some lncRNA targets are located in the center of the network, for example *BAD, PLCB3, MYL6, MEL6B, CACNA2S, CSNK1A1, PRKG1* and *HIF1A*. In the WW sheep, a lncRNA-mRNA co-expression network was constructed for reproductive-related pathways using 29 differentially expressed lncRNAs and 12 target genes. As shown in Fig. 5, some lncRNA targets are located in the center of the network, for example *MAP 3 K11, ANAPC11, FKBP5, FLNB* and *PDPK1*. The network model shows that reproductive-related lncRNA targets are co-expressed with lncRNAs, indicating that lncRNAs and mRNAs are mutually regulated during reproduction.

### RNA-Seq data validation by qPCR

**Fig. 6:**
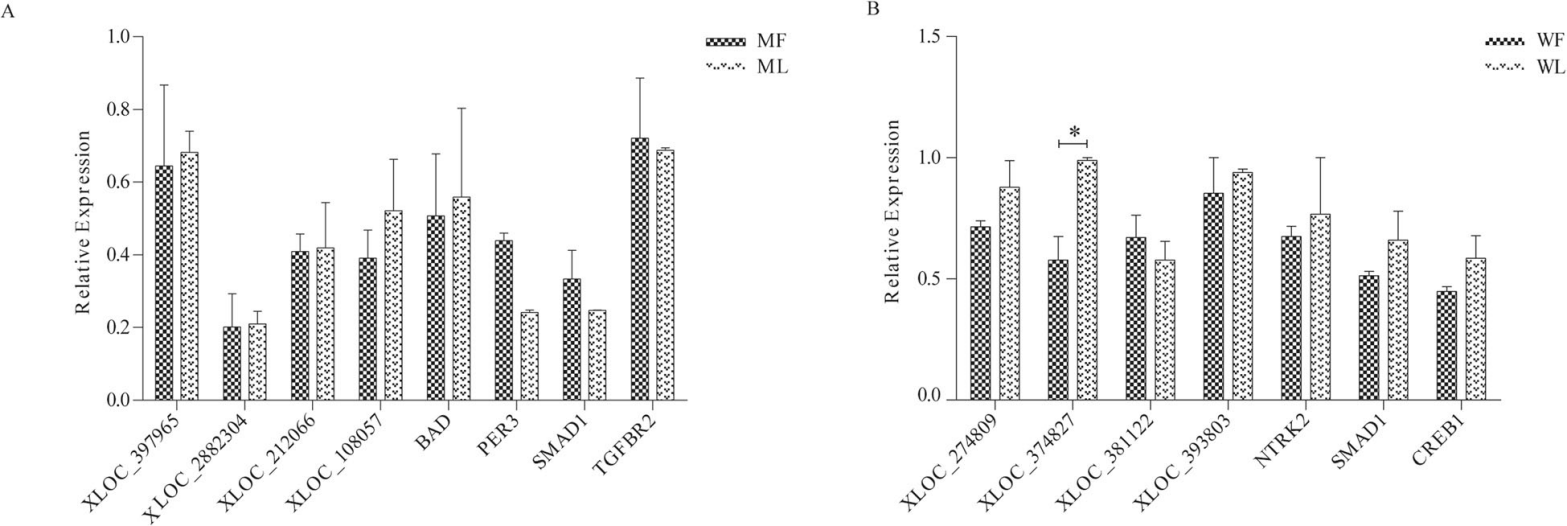
RT–qPCR verification of differentially expressed genes. **a** Verification of the differentially expressed genes in MM sheep. **b** Verification of the differentially expressed genes in WW sheep. Suffixes _F and _L refer to the follicular and luteal phases, respectively

To further validate the sequencing data, 8 differentially expressed lncRNAs and 7 differentially expressed lncRNA target genes were selected to detect expression levels by RT–qPCR (Supplementary Table S7). As shown in Fig. 6, *BAD*, *XLOC_397965, XLOC_2882304, XLOC_212066* and *XLOC_108057* were upregulated in the adrenal glands of the MM sheep and *PER3, SMAD1, TGFBR2* were downregulated in MM sheep. *NTRK2, SMAD1, CREB1*, *XLOC_274809, XLOC_374827, XLOC_381122* and *XLOC_393803* were upregulated in the WW sheep. These results were consistent with the transcriptome data results.

## Discussion

It is known that lncRNAs play an important regulatory role in sheep reproduction. Functional lncRNAs have been identified in the brain, heart, kidney, liver, lung, ovary, skin, white adipose tissue, and pituitary in sheep [13]. In addition, they have also been identified in human, mouse and pig uterine tissues [14, 15]. The adrenal gland, hypothalamus and pituitary were concluded in the hypothalamic–pituitary–adrenal axis (HPA), and the HPA axis interacts with the hypothalamic–pituitary–gonadal (HPG) axis at the brain and pituitary levels to maintain a balance between sheep reproduction and survival. However, little research has been done on lncRNAs and their targets in sheep adrenal glands.

GO annotation and KEGG enrichment analysis indicated that the differentially expressed lncRNA targets were mainly involved in the *Wnt* signaling pathway, *TGF-β* signaling pathway, ovarian steroidogenesis, *MAPK* signaling pathway and circadian rhythm. Analysis of differential lncRNA–mRNA co-expression patterns and functions of target genes revealed that lncRNA might affect the fecundity of sheep by modulating genes associated with the above signaling pathways and biological processes. Among the MM sheep, these pathways were enriched for four differentially expressed lncRNAs (*XLOC_212877, XLOC_357966, 105,614,839, XLOC_154131*) and four lncRNA targets (*TGFBR2, PER3, SMAD1, LHCGR*). In the WW sheep, these pathways were enriched in three differentially expressed lncRNAs (*XLOC_254761, 105,603,287, 105,615,642*) and 3 lncRNA targets (*CREB1, BRAF, NTRK2*).

The HPA axis can be regulated by stress hormone signaling. The paraventricular nucleus in the hypothalamus is activated to release corticotropin releasing hormone (CRH), and CRH stimulates the release of adrenocorticotropic hormone (ACTH). ACTH stimulates the adrenal gland to release glucocorticoids [16, 17]. The HPA can interact with the HPG. For example, estradiol secreted by the ovaries can enhance the activity of the HPA axis. At the level of hypothalamus, CRH inhibits the secretion of gonadotropin releasing hormone (GnRH). In the pituitary, luteinizing hormone levels were significantly reduced in mice chronically exposed to corticosterone [18]. At the level of the adrenal gland, women with congenital adrenal hyperplasia can suffer from impaired fertility or impaired steroid secretion caused by increased androgen levels [19]. High concentrations of glucocorticoids have an inhibitory effect on the activities of GnRH-secreting neurons, pituitary gonadotropins and gonads [12]. In addition, glucocorticoids inhibit thyroid stimulating hormone secretion and reduce the conversion of T3 (inactive thyroxine) to T4 (effective triiodothyronine) during stress. Endogenous thyrotropin-releasing hormone and TSH secretion levels can be inhibited by CRH-induced somatostatin [9]. In sheep, acute stress inhibits the release of LH from the pituitary by inhibiting the synthesis of GnRH and GnRH receptors and promotes functional enhancement of gonadotropin-inhibiting hormone-secreting neurons [20–22]. Clearly, the adrenal gland plays an important role in the HPG.

Cyclic AMP response element binding protein 1 (encoded by *CREB1*) is a member of the CREB family, which is present in different ovarian compartments, including follicular granulosa cells [23–25]. CREB protein concentration increases during sexual maturation and ovarian follicular development [26, 27]. CREB1 can bind to the estrogen receptor alpha to play a key role in chicken sexual maturation [28]. The gene encoding serine/threonine kinase (*BRAF*) is a proto-oncogene that has been identified previously as a candidate target gene in human endometriosis. *CREB1* is a potential transcription factor of *BRAF*. Compared with normal endometrial tissue, the mRNA expression levels of *BRAF* and *CREB1* are significantly upregulated in endometrial tissues of patients with endometriosis; thus, *CREB1* can increase the expression of *BRAF* and regulate cell proliferation by binding directly to *BRAF* in such women [29]. One study determined the role of *CREB1* in mouse granulocytes (mGCs) by knocking down the expression of *CREB1*. It was found that *CREB1* could be regulated by steroid synthesis, cell proliferation, cell cycle, apoptosis and other follicular factors as key regulators of mGCs [30]. Moreover, *XLOC_254761* can transregulate *CREB1*, and *10,563,287* trans-regulated *BRAF*. *CREB1* and *BRAF* are enriched in progesterone-mediated oocyte maturation. These results indicated that those genes enriched in progesterone-mediated oocyte maturation may promote follicular maturation and regulate reproduction in Small-tailed Han sheep.

Using six estrous Hu sheep ovaries and six non-estrous China merino sheep ovaries to identify genes associated with off-season reproduction, the gene encoding neurotrophic receptor tyrosine kinase 2 (*NTRK2*) was found to be differentially upregulated in the ovaries [31]. The *NTRK2* gene encodes the NTRK2 receptor for neurotrophin-4/5 and brain-derived neurotrophic factor leading to oocyte death in late adolescence, loss of follicular tissue and infertility in early adulthood. The preovulatory gonadotropin surge promotes oocyte survival at the onset of reproductive cyclicity by inducing oocyte expression of *NTRK2* in Hu sheep [32]. *105,615,642* can transregulate *NTRK2*, which is enriched in the MAPK signaling pathway. The results suggest that *NTRK2* might regulate follicular survival.

The expression level of the gene encoding luteinizing hormone receptor (*LHCGR*) is low in mural granulosa cells and cumulus cells of antral follicles, and *LHCGR* is induced in granulosa cells by follicle stimulating hormone (FSH). The synthesis of *LHCGR* is mediated by retinoic acid (RA), the demethylation of its promoter region is a key mechanism regulating cell type-specific differentiation during follicular development [33].

Mothers against decapentaplegic homolog 1 (*SMAD1*) is a downstream signal transduction element of bone morphogenetic protein [34, 35], and SMAD1 responds to bone morphogenetic protein 15 (BMP15), which is important for proliferation of granulosa cells in sheep [36]. The transforming growth factor-beta (TGFβ) subfamily is encoded by *TGFB1, TGFB2 and TGFB3*. In mammals, there are seven TGF-1 receptor subtypes (encoded by *TGFBR1*) and five type 2 receptor subtypes (*TGFBR2*) associated with signal transduction ligand of the *TGFβ* superfamily [37, 38]. The TGFβ superfamily ligand initiates an intracellular signaling pathway upon binding to a cell surface receptor complex. In addition, *TGFBR2* in the *TGF-β* signaling pathway were found to be associated with litter size in pigs [39]. There was a significant correlation between litter size in mutant indigenous Chinese Hu sheep and mutations in the three genes encoding the *TGFβ* superfamily (*FecB*, *GDF9*, and *TGFBR2*); moreover, the *FecB*, *GDF9* and *TGFBR2* polymorphisms are considered to be potentially important genetic markers in marker-assisted selection (MAS) strategies to increase litter size in these sheep) [40]. Moreover, here we show that *105,614,839* and *XLOC_212877* cis-regulate *SMAD1* and *TGFBR2*, respectively. These genes are enriched in the TGF-β signaling pathway. The results indicate that *SMAD1* and *TGFBR2* might regulate reproduction in terms of uterine decidual function and litter size.

## Conclusion

In summary, the adrenal gland plays a key role in sheep female reproductive processes. For example, the adrenal gland affects the reproductive of sheep through hypothalamic-pituitary-adrenal axis. These functions of the adrenal gland are achieved by regulating different signaling pathways and related genes. In this study, we screened the differentially expressed lncRNAs (*XLOC_212877*, *XLOC_357966*, *XLOC_154131*, *XLOC_254761*, *105,614,839*, *105,603,287* and *105,615,642*) and differentially expressed lncRNA target genes (*TGFBR2*, *PER3*, *SMAD1*, *LHCGR*, *CREB1*, *BRAF* and *NTRK2*) associated with sheep prolificacy, and constructed networks of interactions between lncRNAs and mRNAs. The lncRNA and mRNAs associated with sheep breeding were enriched by KEGG analysis. The results of the study will help to elucidate the regulatory mechanisms of lncRNAs in sheep reproduction.

## Methods

### Ethics statement

All the animals were authorized by the Science Research Department (in charge of animal welfare issue) of the Institute of Animal Sciences, Chinese Academy of Agricultural Sciences (IAS-CAAS; Beijing, China). In addition, ethical approval of animal survival was given by the animal ethics committee of IAS-CAAS (No. IAAS 2019–449, 18 September 2019).

### Animals and sample collection

Animals were from the core group of Small-tailed Han sheep in the Luxi area of Shandong Province, P. R. China. All were free to eat and drink. We chose healthy nonpregnant sheep aged 2–4 years. Jugular vein blood was collected and the *FecB* mutation was identified by TaqMan assays. A total of 12 sheep (six WW and six MM, respectively) were used for the experiments. All experiments complied with the rules established by the animal ethics committee of IAS-CAAS (No. IAAS 2019–449, 18 September 2019).

All sheep were treated with vaginal sponges (InterAg Co., Ltd., New Zealand) (progesterone 300 mg) for 12 days to synchronize estrus. Three WW and three MM ewes were euthanized (Intravenous pentobarbital (100 mg/kg))on the 50th hours after removing the vaginal sponges, and the adrenal glands were collected (follicular phase; WF and MF, respectively), and of the remaining six sheep (three in each group) were euthanized (Intravenous pentobarbital (100 mg/kg))on the 7th day after sponge removal (luteal phase; WL and ML, respectively) and the adrenal glands were collected. All samples were stored immediately at − 80 °C for total RNA extraction. All experiments complied with the rules established by the the animal ethics committee of IAS-CAAS (No. IAAS 2019–449, 18 September 2019).

### RNA isolation, library preparation and sequencing

Total RNA was extracted from the adrenal glands of all 12 ewes using TRIzol (Thermo Fisher Scientific, Waltham, MA, USA) in accordance with the manufacturer’s instruction. RNA purity was checked using a Nano Photometer® spectrophotometer (IMPLEN, Westlake Village, CA, USA). RNA concentrations were measured using Qubit® RNA Assay kits in a Qubit® 2.0 Fluorometer (Thermo Fisher Scientific, Waltham, MA, USA). RNA integrity was assessed using the RNA Nano 6000 Assay kits of the Bioanalyzer 2100 system (Agilent Technologies, Santa Clara, CA, USA).

The lncRNA library we generated was chain-specific. The method of reverse transcription synthesis of the first strand of cDNA is the same as the New England Biolabs (NEB) general library construction (NEB, Ipswich, MA, USA). The difference is that when the second strand is synthesized, the dTTP in the dNTPs is replaced by dUTP, followed by cDNA end repair, addition of an A tail, ligation sequencing and length screening. Then we used the USER enzyme (New England Biolabs, Inc., Ipswich, MA, USA) to degrade the second strand of U-containing cDNA and performed polymerase chain reaction (PCR) amplification to obtain a library. After the lncRNA library was established, the library preparations were sequenced on an Illumina Hiseq platform (Illumina, San Diego, CA, USA). Raw data of the performed RNA-seq have been recorded in the SRA public database (Accession number: SRP222893).

### Reference genome mapping and transcriptome assembly

Raw reads were obtained by removing reads with an adapter, reads containing > 10% poly-N sequences and low-quality reads. At the same time, Q20, Q30 and GC contents of the clean data were calculated. An index of the reference genome was built using bowtie2 v. 2.2.8 (http://bowtie-bio.sourceforge.net/bowtie2/index.shtml) [41] and paired-end clean reads were aligned to the reference genome *Oar_v3.1* using HISAT2 v. 2.0.4 (https://ccb.jhu.edu/software/hisat2/index.shtml) [42].

The mapped reads of each sample were assembled using StringTie v. 1.3.1 (https://ccb.jhu.edu/software/stringtie/) [42] in a reference-based approach. StringTie uses a novel network flow algorithm as well as an optional de novo assembly step to assemble and quantitate full-length transcripts representing multiple splice variants for each gene locus.

### Identification of potential lncRNA candidates

Potential lncRNA candidates were identified as follows: selecting transcripts with exon number ≥ 2 and transcript length > 200 nt. The transcripts obtained by combining each sample using Cuffdiff v. 2.1.1 (https://software.broadinstitute.org/cancer/software/genepattern/modules/docs/Cuffcompare/8) [43], and transcripts with uncertain chain direction were removed to obtain complete transcriptome information.

The intersection of transcripts with no coding potential in these software analysis results was used as the lncRNA data set. CNCI v. 2.0 (https://github.com/www-bioinfo-org/CNCI) [44], CPC v. 2.81 (http://cpc.cbi.pku.edu.cn/) [45] and PFAM v. 1.3 (https://pfam.xfam.org) [46] were used to evaluate the protein coding potential of the transcript and the results of these software as the end result.

### Analysis of differentially expressed genes

Gene expression estimation using the FPKM value expected number of Fragments Per Kilobase of transcript sequence per million base pairs sequenced) eliminates the effect of sequencing depth and gene length on fragment counts. Different types of transcripts (lncRNAs and mRNAs) were analyzed for differences using Cuffdiff v. 2.1.1 (https://software.broadinstitute.org/cancer/software/genepattern/modules/docs/Cuffdiff/7) [43]. LncRNAs and mRNAs with *P* values < 0.05 were considered as differentially expression between the two groups of data.

### GO annotation and KEGG pathway enrichment analysis of differentially expressed genes

The GO is an international standard system including molecular functions, biological processes, and cellular components for classifying gene function. Pathway enrichment analysis can identify major metabolic pathways and signaling pathways enriched by differentially expressed genes. Enrichment analysis was performed on each Pathway in KEGG using a hypergeometric test. The calculated *P* value and 0.05 being defined as the significant threshold, the genes were screened and enriched for the pathways. Next, the significance of the pathway enrichment analysis was corrected by FDR, and the corrected *P*-value (Q-value) was obtained. Differentially expressed genes were further studied using the GO and KEGG databases to study the functions of the genes and identify the pathways in which they participate. If a P value was≤0.05, enrichment was considered significant.

### GO annotation and KEGG pathway enrichment analysis of differentially expressed lncRNA targets

Differentially expressed lncRNAs regulate the target genes by cis-regulating nearby genes and trans-regulating distal protein-coding genes. Here, protein-coding genes with a distance of less than 100 Kb were assumed to be the cis-target genes, and Pearson correlation coefficients with the lncRNAs of > 0.95 were assumed to represent the trans-target genes [47]. Statistical enrichment of differentially expressed lncRNA target genes in the GO annotation and KEGG pathways were evaluated, and *P* value≤0.05 was considered significant enrichment.

### Construction of lncRNA/mRNA networks

To infer the functions of differentially expressed lncRNAs and their target genes in sheep prolificacy, we constructed a network based on mRNAs and lncRNAs in Cytoscape v. 3.1.1 (https://cytoscape.org).

### Reverse transcription (RT)–qPCR verification

RT–qPCR was used to verify the expression levels of differentially expressed lncRNAs and their targets. About 0.1 μg of RNA was used per sample and this was reverse transcribed into cDNA using RT reagents (Thermo Fisher Scientific, Waltham, MA, USA). All experiments were performed in triplicate, and β-actin was used as an internal reference to normalize target gene expression. The qPCR was performed on a LightCycler 480II (Roche, Basel, Sweden) using SYBR Premix Ex Taq II. The procedure involved 40 cycles of pre-denaturation at 95 °C for 5 s; denaturation at 95 °C for 5 s, 60 °C for 30 s. After the reaction was completed, melting curve analysis was performed. The relative expression level of the target genes was calculated by the 2^–ΔΔCt^ method [48]. The lncRNAs and target gene primers are shown in Supplemental Table S1.

### Statistical analyses

All data are presented as the mean ± SD. Student’s *t* tests were performed to compare means, and *P* < 0.05 was considered statistically significant.

## Supplementary information

**Additional file 1: Table S1.** Differentially expressed mRNAs and lncRNAs between follicular phase and luteal phase in MM sheep.

**Additional file 2: Table S2.** Differentially expressed mRNAs and lncRNAs between follicular phase and luteal phase in WW sheep.

**Additional file 3: Table S3.** The differentially expressed mRNAs GO enrichment analyses between follicular phase and luteal phase in MM sheep.

**Additional file 4: Table S4.** The differentially expressed mRNAs GO enrichment analyses between follicular phase and luteal phase in WW sheep.

**Additional file 5: Table S5.** KEGG enrichment analysis of differentially expressed mRNAs between follicular phase and luteal phase in MM and WW sheep.

**Additional file 6: Table S6.** KEGG enrichment analysis of differentially expressed lncRNAs between follicular phase and luteal phase in MM and WW sheep.

**Additional file 7 : Table S7.** Details of primer sequences and expected product sizes of lncRNAs and lncRNA targets used for RT–qPCR.

## Data Availability

The following information was supplied regarding data availability: Data is available at the Sequence Read Archive (SRA222893).

## Abbreviations

LncRNAs: Long noncoding RNAs
MAPK: The mitogen-activated protein kinase cascade
HPA: The hypothalamic–pituitary–adrenal axis
HPG: The hypothalamic–pituitary–gonadal axis
CRH: Corticotropin releasing hormone
ACTH: The release of adrenocorticotropic hormone
GnRH: Gonadotropin releasing hormone
*CREB1*: Cyclic AMP response element binding protein 1
*BRAF*: Serine/threonine kinase
mGCs: Mouse granulocytes
*NTRK2*: Neurotrophic receptor tyrosine kinase 2
*LHCGR*: Luteinizing hormone receptor
FSH: Follicle stimulating hormone
*SMAD1*: Mothers against decapentaplegic homolog 1
BMP15: Bone morphogenetic protein 15
*TGFBR1*: TGF-1 receptor subtypes
*TGFBR2*: Type 2 receptor subtypes
MAS: Marker-assisted selection

## References

[1] Di R, Chu MX, Li YL, Zhang L, Fang L, Feng T, Cao GL, Chen HQ, Li XW (2012). Predictive potential of microsatellite markers on heterosis off ecundity in crossbred sheep. Mol Biol Rep.

[2] Chu M, Jia L, Zhang Y, Jin M, Chen H, Fang L, Di R, Cao G, Feng T, Tang Q, Ma Y, Li K (2011). Polymorphisms of coding region of BMPR-IB gene and their relationship with litter size in sheep. Mol Biol Rep.

[3] Miao X, Luo Q, Zhao H, Qin X (2016). Co-expression analysis and identification of fecundity-related long non-coding RNAs in sheep ovaries. Sci Rep.

[4] Miao X, Luo Q, Zhao H, Qin X (2017). An integrated analysis of miRNAs and methylated genes encoding mRNAs and lncRNAs in sheep breeds with different fecundity. Front Physiol.

[5] Yang H, Wang F, Li F, Ren C, Pang J, Wan Y, Wang Z, Feng X, Zhang Y (2018). Comprehensive analysis of long non-coding RNA and mRNA expression patterns in sheep testicular maturation. Biol Reprod.

[6] Zheng J, Wang Z, Yang H, Yao X, Yang P, Ren C, Wang F, Zhang Y. Pituitary transcriptomic study reveals the differential regulation of lncRNAs and mRNAs related to prolificacy in different FecB genotyping sheep. Genes (Basel). 2019. 10.3390/genes10020157. 10.3390/genes10020157 PMC6410156 30781725

[7] Feng X, Li FZ, Wang F, Zhang GM, Pang J, Ren CF, Zhang TT, Yang H, Wang ZY, Zhang YL. Genome-wide differential expression profiling of mRNAs and lncRNAs associated with prolificacy in Hu sheep. Biosci Rep. 2018. 10.1042/BSR20171350. 10.1042/BSR20171350 PMC5920141 29439142

[8] Jimena P, Castilla JA, Peran F, Ramirez JP, Jr FV, Molina R, Vergara F, Herruzo A. Adrenal hormones in human follicular fluid. Acta Endocrinol, 1992;127(5):403. 10.1530/acta.0.1270403 1471451

[9] Magiakou MA, Mastorakos G, Webster E, Chrousos GP (1997). The hypothalamic-pituitary-adrenal axis and the female reproductive system. Ann N Y Acad Sci.

[10] Gao Y, Chen F, Kong QQ, Ning SF, Yuan HJ, Lian HY, Luo MJ, Tan JH (2016). Stresses on female mice impair oocyte developmental potential: effects of stress severity and duration on oocytes at the growing follicle stage. Reprod Sci.

[11] Yuan HJ, Han X, Nan H, Guo LW, Shuai G, Juan L, Min G, Tian JH (2016). Glucocorticoids impair oocyte developmental potential by triggering apoptosis of ovarian cells via activating the Fas system. Sci Rep.

[12] Whirledge S, Cidlowski JA (2017). Glucocorticoids and reproduction: traffic control on the road to reproduction. Trends Endocrinol Metab.

[13] Bakhtiarizadeh MR, Hosseinpour B, Arefnzhad B, Shamabadi N, Salami SA (2016). In silico prediction of long intergenic non-coding RNAs in sheep. Genome.

[14] Zhou C, Zhang T, Liu F, Zhou J, Ni X, Huo R, Shi R (2015). The differential expression of mRNAs and long noncoding RNAs between ectopic and eutopic endometria provides new insights into adenomyosis. Mol BioSyst.

[15] Wang Y, Xue S, Liu X, Hu T, Qiu X, Zhang J, Lei M (2016). Analyses of long non-coding RNA and mRNA profiling using RNA sequencing during the pre-implantation phases in pig endometrium [J]. Sci Rep.

[16] Herbison AE, Robert P, Jean-Remi P, Mora JM, Hurst PR (2008). Gonadotropin-releasing hormone neuron requirements for puberty, ovulation, and fertility. Endocrinology.

[17] Lehman MN, Hileman SM, Goodman RL (2015). Neuroanatomy of the kisspeptin signaling system in mammals: comparative and developmental aspects. Adv Exp Med Biol.

[18] Rivier C, Vale W (1984). Corticotropin-releasing factor (CRF) acts centrally to inhibit growth hormone secretion in the rat. Endocrinology.

[19] Gomes LG, Bachega TASS, Mendonca BB (2019). Classic congenital adrenal hyperplasia and its impact on reproduction. Fertil and Steril.

[20] Dobson H, Fergani C, Routly JE, Smith RF (2012). Effects of stress on reproduction in ewes. Anim Reprod Sci.

[21] Ciechanowska M, Lapot M, Antkowiak B, Mateusiak K, Paruszewska E, Malewski T, Paluch M, Przekop F (2016). Effect of short-term and prolonged stress on the biosynthesis of gonadotropin-releasing hormone (GnRH) and GnRH receptor (GnRHR) in the hypothalamus and GnRHR in the pituitary of ewes during various physiological states. Anim Reprod Sci.

[22] Clarke IJ, Bartolini D, Conductier G, Henry BA (2016). Stress increases gonadotropin inhibitory hormone cell activity and input to gnRH cells in ewes. Endocrinology.

[23] Gubbay O, Rae MT, McNeilly AS, Donadeu FX, Zeleznik AJ, Hillier SG (2006). cAMP response element-binding (CREB) signalling and ovarian surface epithelial cell survival. J Endocrinol.

[24] Pei JH, Fujimoto Y, Yamauchi N, Hattori MA (2006). Real-time monitoring of cAMP response element binding protein signaling in porcine granulosa cells modulated by ovarian factors. Mol Cell Biochem.

[25] Suman R, Androulla E, Zara J, Laura P, Maon HD (2013). Metformin inhibits follicle-stimulating hormone (FSH) action in human granulosa cells: relevance to polycystic ovary syndrome. J Clin Endocrinol Metab.

[26] Natalie YO, Noa S, Naomi MB, Sarah E, Moriah K, Manna PR, Stocco DM, Joseph O (2009). Transcription of steroidogenic acute regulatory protein in the rodent ovary and placenta: alternative modes of cyclic adenosine 3′, 5′-monophosphate dependent and independent regulation. Endocrinology.

[27] Sirotkin AV, Gupta K, Kapoor R, Dwivedi A (2019). MicroRNA-145 targets Smad1 in endometrial stromal cells and regulates decidualization in rat. J Mol Med (Berl).

[28] Guo M, Li Y, Chen Y, Guo X, Yuan Z, Jiang Y (2018). Genome-wide mapping of estrogen receptor α binding sites by ChIP-seq to identify genes related to sexual maturity in hens. Gene.

[29] Lv X, Wang D, Ma Y, Long Z (2018). Analysis of the oncogene BRAF mutation and the correlation of the expression of wild-type BRAF and CREB1 in endometriosis. Int J Mol Med.

[30] Zhang P, Wang J, Lang H, Wang W, Liu X, Liu H, Tan C, Li X, Zhao Y, Wu X (2018). Knockdown of CREB1 promotes apoptosis and decreases estradiol synthesis in mouse granulosa cells. Biomed Pharmacother.

[31] Lei C, Liu K, Zhao Z, Blai HT, Peng Z, Li D, Ma RZ (2012). Identification of sheep ovary genes potentially associated with off-season reproduction. J Genet Genomics.

[32] Dorfman MD, Cecilia GR, Zefora A, Bredford K, Alejandro L, Dissen GA, Juan MC, David GG, Francisco G, Baoji X (2014). Loss of Ntrk2/Kiss1r signaling in oocytes causes premature ovarian failure. Endocrinology.

[33] Kawai T, Richards JS, Shimada M (2018). The cell type–specific expression of Lhcgr in mouse ovarian cells: evidence for a DNA-demethylation–dependent mechanism. Endocrinology.

[34] Caterina C, Tripurani SK, Large MJ, Edson MA, Creighton CJ, Hawkins SM, Kovanci E, Kaartinen V, Lydon JP, DeMayo FJ, Matzuk MM (2013). Activin-like kinase 2 functions in peri-implantation uterine signaling in mice and humans. PLoS Genet.

[35] Kim MR, Park DW, Lee JH, Choi DS, Hwang KJ, Ryu HS, Min CK (2005). Progesterone-dependent release of transforming growth factor-beta1 from epithelial cells enhances the endometrial decidualization by turning on the Smad signalling in stromal cells. Mol Hum Reprod.

[36] Moore RK, Otsuka F, Shimasaki S (2003). Molecular basis of bone morphogeneric protein-15 signaling in gramulosa cells. J Biol Chem.

[37] Claire G, Kemp CF, Knight PG (2004). Bone morphogenetic protein (BMP) ligands and receptors in bovine ovarian follicle cells: actions of BMP-4, −6 and −7 on granulosa cells and differential modulation of Smad-1 phosphorylation by follistatin. Reproduction.

[38] Knight PG, Glister C (2006). TGF-beta superfamily members and ovarian follicle development. Reproduction.

[39] Li XJ, Ye JW, Han X, Qiao RM, Li XL, Lv G, Wang KJ. Whole-genome sequencing identifies potential candidate genes for reproductive traits in pigs. Genomics. 2019. 10.1016/j.ygeno.2019.01.014. 10.1016/j.ygeno.2019.01.014 30707936

[40] Wang Q, Wang N, Cai R, Zhao F, Xiong Y, Li X, Wang A, Lin P, Jin Y (2017). Genome-wide analysis and functional prediction of long non-coding RNAs in mouse uterus during the implantation window. Oncotarget.

[41] Langmead B, Salzberg SL (2012). Fast gapped-read alignment with bowtie 2. Nat Methods.

[42] Pertea M, Kim D, Pertea GM, Leek JT, Salzerg SL (2016). Transcript-level expression analysis of RNA-seq experiments with HISAT, StringTie and Ballgown. Nat Protoc.

[43] Trapnell C, Wiliams BA, Pertea G, Mortazavi A, Kwan G, Ven Baren MJ, Salzberg SL, Wold BJ, Pachter L (2010). Transcript assembly and quantification by RNA-Seq reveals unannotated transcripts and isoform switching during cell differentiation. Nat Biotechnol.

[44] Sun L, Luo H, Bu D, Zhao G, Yu K, Zhang C, Liu Y, Chen R, Zhao Y (2013). Utilizing sequence intrinsic composition to classify protein-coding and long non-coding transcripts. Nucleic Acids Res.

[45] Kang YJ, Yang DC, Kong L, Hou M, Meng YQ, Wei L, Gao G (2017). CPC2: a fast and accurate coding potential calculator based on sequence intrinsic features. Nucleic Acids Res.

[46] Bateman A, Birney E, Cerruti L, Durbin R, Etwiller L, Eddy SR, Griffiths-Jones S, Howe KL, Marshall M, Sonnhammer EL (2002). The Pfam protein families database. Nucleic Acids Res.

[47] Alessandro F, Irene B (2014). Long non-coding RNAs: new players in cell differentiation and development. Nat Rev Genet.

[48] Schmittgen TD, Libak KJ (2008). Analyzing real-time PCR data by the comparative C(T) method. Nat Protoc.

